# Immunogenicity and Safety of a Single Booster Dose of Sabin-Strain Inactivated Poliovirus Vaccine in Adolescents and Adults: A Phase IV, Open-Label Trial

**DOI:** 10.3390/vaccines14090808

**Published:** 2026-09-14

**Authors:** Xiaoshu Zhang, Weixiao Han, Jianfeng Wang, Yu Tang, Yanan Ji, Liping Lu, Junxia Zuo, Yijing Nie, Yajing Yao, Jun Li, Dan Yu, Jing An

**Affiliations:** 1 Gansu Provincial Center for Disease Control and Prevention, Gansu Provincial Academy of Preventive Medicine, Lanzhou 730300, China; zhxs222@126.com (X.Z.); 18418669439@163.com (Y.J.); 2 Sinovac Biotech Co., Ltd., Beijing 100085, China; hanwx@sinovac.com (W.H.); wangjianfeng@sinovac.com (J.W.); nieyijing9448@sinovac.com (Y.N.); yaoyj2640@sinovac.com (Y.Y.); lijun@sinovac.com (J.L.); 3 Zhangye Municipal Center for Disease Control and Prevention, Zhangye 734000, China; zycdcepi@163.com; 4 Longxi County Center for Disease Control and Prevention, Longxi County Health Supervision Institute, Dingxi 748100, China; 13830259573@163.com

**Keywords:** poliovirus, inactivated vaccine, sIPV, adolescents, adults, immunogenicity, safety, China

## Abstract

Background: In China, the current national immunisation schedule extends only to 4 years of age, and no polio booster dose is recommended for adolescents or adults, with data on immunogenicity and safety in these populations lacking. This phase IV trial evaluated the immunogenicity and safety of a single booster dose of Sabin-strain inactivated poliovirus vaccine (sIPV) in Chinese adolescents and adults. Methods: This phase IV, open-label trial enrolled 120 healthy participants, comprising 60 adolescents aged 7–17 years and 60 adults aged 18–50 years, who received a single booster dose of sIPV. Participants were enrolled and vaccinated in Gansu Province, China, between 21 October and 1 November 2025. Immunogenicity was assessed by neutralising antibody titres against poliovirus types 1, 2, and 3 before and 30 days post-vaccination. Safety was evaluated through monitoring of adverse events throughout the 30-day follow-up period. Results: Following a single sIPV booster dose, seropositivity rates reached 100% for all three poliovirus serotypes in both age groups. Post-vaccination geometric mean titres (GMTs) for types 1, 2, and 3 were 1748.29, 3961.77, and 2089.08 in adolescents, and 1902.58, 4434.39, and 2256.16 in adults, respectively, with no significant between-group differences. Seroconversion rates for type 3 were significantly higher in adults than in adolescents (98.33% vs. 86.67%, *p* = 0.032). Geometric mean increases (GMIs) were significantly higher in adults for type 1 (72.12 vs. 28.10, *p* = 0.008) and type 3 (316.14 vs. 45.56, *p* < 0.001). The vaccine was well tolerated; vaccine-related adverse events were reported in 17.50% of participants, all mild to moderate, with no vaccine-related serious adverse events. Conclusions: A single booster dose of sIPV induced robust neutralising antibody responses across all three poliovirus serotypes in both adolescents and adults, with a favourable safety profile. Adults with lower baseline immunity derived proportionally greater fold-increases in antibody titres.

## 1. Introduction

Poliomyelitis is an acute infectious disease caused by poliovirus that primarily affects children and may result in irreversible paralysis or even death. Since the launch of the Global Polio Eradication Initiative (GPEI), global poliomyelitis incidence has declined dramatically as a result of sustained high vaccination coverage worldwide [1]. Although wild poliovirus types 2 and 3 were declared eradicated in 2015 and 2019, respectively, type 1 remains endemic in Afghanistan and Pakistan, both of which border China, making importation and subsequent outbreaks a persistent concern for China [2,3].

Oral poliovirus vaccine (OPV), despite its pivotal role in reducing transmission, carries inherent risks of vaccine-associated paralytic poliomyelitis (VAPP) and vaccine-derived poliovirus (VDPV) outbreaks [4]. These limitations have driven the transition toward inactivated poliovirus vaccine (IPV) in national immunization programs [2]. The WHO Strategic Advisory Group of Experts on Immunization (SAGE) has explicitly stated that IPV use represents a long-term strategy [5]. In China, following multiple schedule revisions, the current national childhood immunisation programme (2021 version) prescribes one IPV dose at 2 months and 3 months of age, followed by one bOPV dose at 4 months and 4 years of age [6].

Most immunogenicity studies on IPV have focused on infants and young children, with relatively limited data available for older populations. Among the few studies that have evaluated IPV booster responses in adults, evidence from Cuba and Japan has demonstrated that a single dose effectively elevates neutralising antibody levels against all three serotypes, with sustained protection documented at 21 months post-vaccination in the Cuban cohort [7,8]. A Japanese catch-up study further showed that among adults with one or two prior tOPV doses, a supplemental IPV dose elicited seropositivity rates exceeding 98.4% for all three serotypes, with geometric mean increases ranging from 17.7 to 53.9 [8].

Despite these encouraging findings, recommendations for adult poliovirus booster vaccination vary substantially across countries. In France, the national immunization program recommends booster doses against diphtheria, tetanus, poliomyelitis, and pertussis at 25, 45, and 65 years of age [9]. In the United States, following a VDPV2 case in New York in 2022, the Advisory Committee on Immunization Practices (ACIP) updated its recommendations to include a three-dose IPV primary series for adults with incomplete vaccination and a single booster dose for those at increased exposure risk [10]. In Italy, a systematic review and meta-analysis revealed that the proportion of individuals lacking neutralising antibodies against PV1, PV2, and PV3 was 6.4%, 5.3%, and 13.0%, respectively, with antibody titres declining notably with age, particularly during adolescence, which supported the introduction of a fifth IPV-containing dose at 12–18 years of age into the national schedule [11,12]. In China, however, the current vaccination schedule does not extend beyond 4 years of age, and no booster dose is recommended for adolescents or adults. Vaccination records for many individuals aged 50 years and younger remain difficult to ascertain due to the phased rollout of the immunisation information system, and although most of this population is presumed to have received primary vaccination given decades of routine immunisation, the need for and immunogenicity of a booster dose in these age groups have not been systematically evaluated.

Given the favourable safety profile of sIPV established in multiple clinical trials, which showed no significant difference compared with the alum-adjuvanted IPV vaccine (Sanofi Pasteur) licensed for adult use [13,14,15,16], we conducted this phase IV study to evaluate the immunogenicity and safety of a single booster dose of sIPV in adolescents (7–17 years) and adults (18–50 years). The findings are expected to inform the evidence base for potential expansion of sIPV use to adolescent and adult populations in China.

## 2. Methods

### 2.1. Vaccines

The investigational product was Sabin strain inactivated poliomyelitis vaccine (Vero cells) (sIPV), manufactured by Sinovac Biotech Co., Ltd. (Beijing, China), with batch number KA202411038. The vaccine received marketing authorisation in China on 12 July 2021 (Drug Registration Certificate No. 2021S00781). It was supplied in prefilled syringes (0.5 mL per dose, each containing 15 D-antigen units of type 1, 45 D-antigen units of type 2, and 45 D-antigen units of type 3 poliovirus antigens). Each participant received a single 0.5 mL dose via intramuscular injection into the deltoid muscle. The vaccine was packaged individually in separate boxes, each containing one prefilled syringe. All study vaccines were stored at 2 °C to 8 °C protected from light and were not frozen. Vaccines were transported to the study sites under cold-chain conditions (2–8 °C), and temperature logs were maintained throughout storage. Any vaccines with damaged packaging, suspected cold-chain breach, or frozen storage were not used.

### 2.2. Study Design and Participants

This was an open-label, phase IV study conducted at three sites in China, including Chengguan District (Lanzhou City), Longxi County (Dingxi City), and Zhangye City, with participant enrolment and vaccination conducted between 21 October and 1 November 2025. The trial was registered at ClinicalTrials.gov (NCT07354269) and monitored by Sinovac Biotech Co., Ltd. The study participants were healthy adolescents aged 7–17 years and adults aged 18–50 years. All eligible participants received a single 0.5 mL dose of sIPV on day 0, with immunogenicity and safety assessments conducted up to 30 days post-vaccination.

Inclusion criteria included: (1) age 7–50 years; (2) written informed consent obtained from all participants aged ≥ 18 years, and from both participants and their legal guardians for those aged 7–17 years; (3) provision of valid identification; (4) willingness and ability to comply with all scheduled visits, sample collections, vaccination, and other study procedures, and to remain contactable throughout the study; and (5) use of effective contraception from enrolment through 30 days post-vaccination for participants of childbearing potential and their partners, with avoidance of egg or sperm donation during the study period. Exclusion criteria included: (1) a history of poliomyelitis or poliovirus infection; (2) uncontrolled chronic disease or history of serious illness, including but not limited to cardiovascular, haematological, hepatic, renal, digestive, or respiratory diseases, malignancies, or major organ transplantation; (3) autoimmune or immunodeficiency disorders, including but not limited to systemic lupus erythematosus, ankylosing spondylitis, autoimmune thyroid disease, asplenia, functional asplenia, or HIV infection; (4) coagulation abnormalities; (5) severe congenital malformations, genetic defects, or malnutrition; (6) history of severe neurological disorders (epilepsy, seizures, or convulsions, excluding history of febrile seizures) or psychiatric illness, or family history of psychiatric disorders; (7) acute illness or acute exacerbation of chronic disease within 3 days, or known or suspected active infection; (8) receipt of immunosuppressive or other immunomodulatory therapy for ≥14 days within the past 6 months (prednisone ≥ 20 mg/day for adults or ≥2 mg/kg/day for individuals aged <18 years, or equivalent), cytotoxic therapy, or planned receipt of such therapies during the study; (9) receipt of immunoglobulin or other blood products within the past 6 months, or planned receipt during the study; (10) receipt of other investigational drugs or vaccines within 30 days, or planned receipt during the study; (11) receipt of live-attenuated or nucleic acid vaccines within 14 days, or subunit or inactivated vaccines within 7 days before study vaccination; (12) known allergy to any component of the study vaccine (inactivated poliovirus, Medium 199, glycine, sodium chloride, potassium chloride, calcium chloride, magnesium phosphate, disodium hydrogen phosphate, sodium dihydrogen phosphate); (13) pregnancy, lactation, or planned pregnancy within 3 months after vaccination; (14) axillary temperature > 37 °C on the day of vaccination; (15) abnormal physical examination on the day of vaccination; (16) injection site conditions such as skin damage, inflammation, ulceration, rash, or scarring that could interfere with vaccination or local reaction assessment; or (17) any other condition deemed unsuitable for participation by the investigator.

The sample size was calculated using the formula for a single proportion:

$$N=(Z^{2}\times P\times (1-P))/\varepsilon^{2},$$

with Z = 1.96, *p* = 0.984 (the seropositivity rate reported in a Japanese catch-up study of IPV booster in adults [8]), and ɛ = 0.04. This yielded 38 participants per group. To allow for a potential 10% drop-out rate and to ensure adequate precision for secondary endpoints and subgroup analyses, the target enrolment was set at 60 participants per age group.

### 2.3. Procedures

Screening was conducted within 14 days before vaccination or on the day of vaccination. Investigators responsible for informed consent underwent training to ensure that study information was communicated in clear, understandable language. After providing written informed consent, potential participants were assigned a screening number and assessed against the predefined inclusion and exclusion criteria. Eligible participants were enrolled and allocated a study number sequentially (A01–A60 for adolescents aged 7–17 years; B01–B60 for adults aged 18–50 years). On the day of vaccination, demographic characteristics, medical history, and concomitant medications were recorded; physical examination and vital signs were assessed, and pregnancy testing was performed for females of childbearing potential. Eligibility was reconfirmed against all inclusion and exclusion criteria before vaccination. Participants then received a single dose of sIPV, followed by a 30 min observation period for immediate adverse events (AEs). Any AEs or serious adverse events (SAEs) observed during this period were recorded. AEs were defined as any untoward medical occurrence in participants following vaccination, irrespective of causal relationship. SAEs were defined as any AE that resulted in death, was life-threatening, required or prolonged hospitalization, caused persistent or significant disability/incapacity, constituted a congenital anomaly/birth defect, or was otherwise considered medically important.

Diary cards were provided on the day of vaccination to record solicited and unsolicited AEs from day 0 to day 7, and unsolicited AEs from day 8 to day 30. On day 8 post-vaccination, a telephone contact was made to review diary card entries and collect information on AEs and SAEs. A face-to-face visit was scheduled on day 30 to verify and retrieve diary cards, collect blood samples for immunogenicity assessment, and document AEs and SAEs. Blood samples (2.5–3.0 mL) were collected before vaccination and at 30 days post-vaccination. Serum was separated and sent to Sinovac Biotech Co., Ltd., for detection of neutralising antibodies against poliovirus types 1, 2, and 3 using a microneutralisation assay. The antibody testing assay used in this study is a micro-neutralization assay with cytopathic effect (CPE) as the endpoint. Neutralizing antibodies against Sabin-strain poliovirus types 1, 2 and 3 were measured by a CPE-endpoint micro-neutralization assay. Serum samples were heat-inactivated at 56 °C and serially two-fold diluted, then incubated with Sabin-strain virus (100 CCID50/0.05 mL) at 35 °C for 3 h. Hep-2 cells were added and cultured for 7 days, followed by microscopic observation for cytopathic effect. The neutralizing antibody titer, defined as the reciprocal of the serum dilution that protects 50% of cell culture wells from CPE, was calculated by the Karber method based on the number of CPE-positive wells across serial serum dilutions. This assay has a limit of detection of 1:4, and is applied for evaluating immune sera induced by sIPV vaccine.

### 2.4. Outcomes

The primary immunogenicity endpoints were the seroconversion rates of neutralising antibodies against poliovirus types 1, 2, and 3 at 30 days post-vaccination in adolescents aged 7–17 years and adults aged 18–50 years, respectively. Seroconversion was defined as a pre-vaccination antibody titre < 1:8 with a post-vaccination titre ≥ 1:8, or a ≥4-fold increase from pre-vaccination levels for those with a baseline titre ≥ 1:8. Secondary immunogenicity endpoints included seropositivity rates, geometric mean titres (GMTs), and geometric mean increases (GMIs) for each serotype at 30 days post-vaccination. Seropositivity was defined as a neutralising antibody titre ≥ 1:8. Safety endpoints included the incidence of AEs and serious adverse events (SAEs) throughout the 30-day post-vaccination period.

### 2.5. Statistical Analyses

Demographic and baseline characteristics were summarised using descriptive statistics. Continuous variables were presented as means and standard deviations (SDs), and categorical variables as frequencies and percentages. Between-group comparisons for continuous variables were performed using the independent-samples t-test, and for categorical variables using the Chi-square test or Fisher’s exact test, as appropriate. For each poliovirus serotype, seroconversion rates and seropositivity rates at 30 days post-vaccination were calculated with their two-sided 95% confidence intervals (CIs) using the Clopper–Pearson method. Seroconversion rates and seropositivity rates were compared between the two age groups using the Chi-square test or Fisher’s exact test. Geometric mean titres (GMTs) and geometric mean increases (GMIs) were calculated with 95% CIs. GMTs and GMIs were log-transformed to approximate normality before analysis, and between-group differences were assessed using the independent-samples t-test on the log-transformed values. AEs were coded using the Medical Dictionary for Regulatory Activities (MedDRA) and summarised by system organ class (SOC) and preferred term. The incidence of vaccine-related AEs, solicited local and systemic adverse reactions, and serious SAEs was tabulated. All statistical tests were two-sided, and a *p*-value of <0.05 was considered statistically significant. Statistical analyses were performed using SAS version 9.4 (SAS Institute, Cary, NC, USA).

## 3. Results

### 3.1. Study Participants

**Figure 1:**
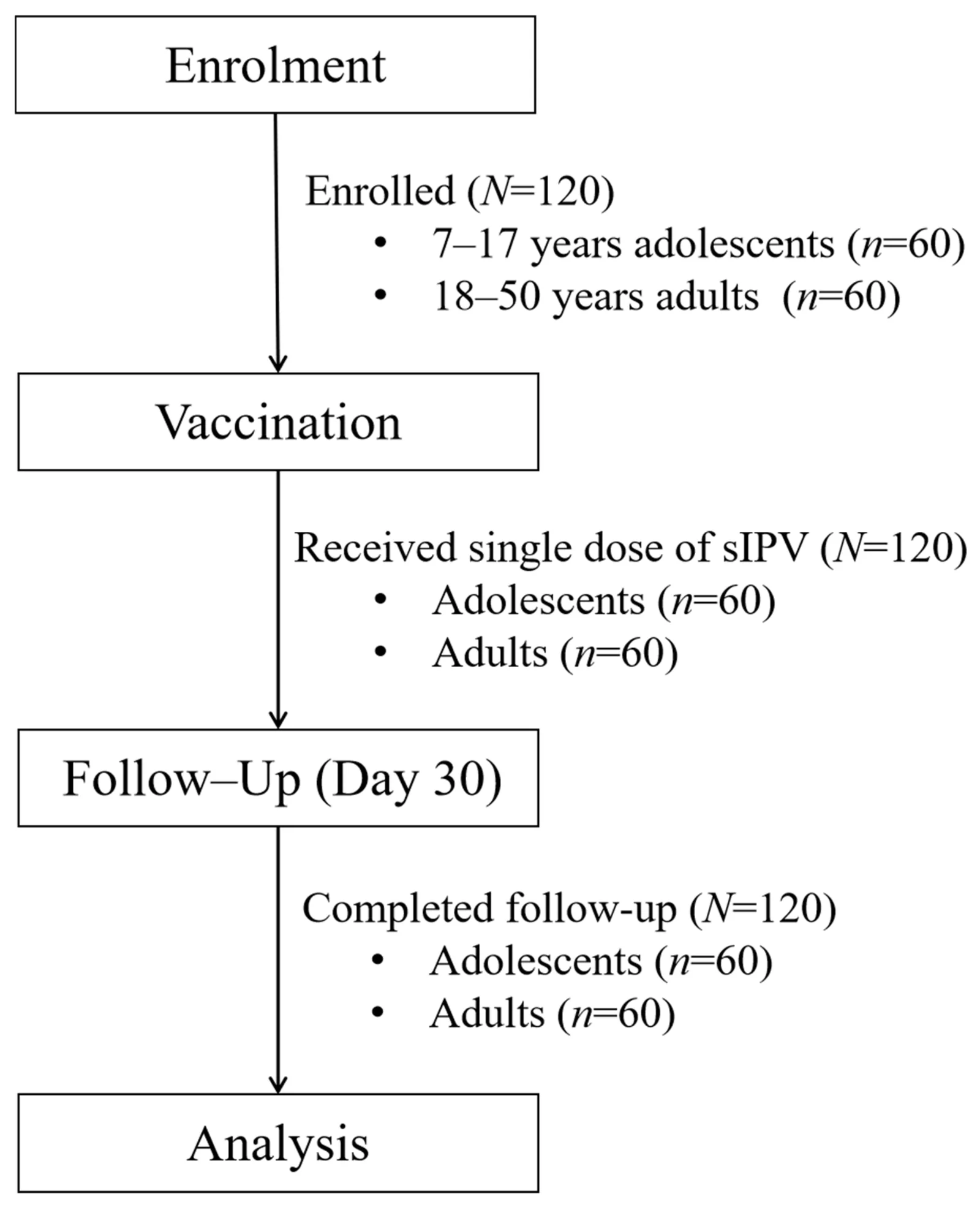
The flowchart of this study.

A total of 120 healthy participants were enrolled, comprising 60 adolescents aged 7–17 years and 60 adults aged 18–50 years. All 120 participants (100%) completed the scheduled vaccination and attended the 30-day post-vaccination follow-up visit (Figure 1). The baseline demographic and clinical characteristics of the study population are summarised in Table 1. The mean age was 10.0 years (SD 2.5) in the adolescent group and 37.0 years (SD 7.4) in the adult group. The proportion of male participants was 50.0% in the adolescent group and 31.7% in the adult group. The majority of participants were of Han ethnicity (98.33% in each group). Baseline anthropometric measurements and axillary temperatures were within normal ranges.

### 3.2. Immunogenicity

Following a single booster dose of sIPV, robust neutralising antibody responses were elicited against all three poliovirus serotypes in both age groups. Seroconversion rates for poliovirus type 1 did not differ significantly between adolescents and adults (88.33% vs. 91.67%, *p* = 0.543), nor did those for type 2 (98.33% in both groups, *p* = 0.999). However, for type 3, the rate was significantly higher in adults than in adolescents (98.33% vs. 86.67%, *p* = 0.032). Before vaccination, baseline seropositivity rates and GMTs for poliovirus type 2 were similar between the two groups (*p* > 0.05). For type 1, adolescents had significantly higher baseline seropositivity rates (91.67% vs. 75.00%, *p* = 0.014) and GMTs (62.21 vs. 26.38, *p* = 0.002) than adults. For type 3, adolescents also showed significantly higher baseline seropositivity (78.33% vs. 43.33%, *p* < 0.001) and GMTs (45.85 vs. 7.14, *p* < 0.001). After vaccination, seropositivity rates reached 100% for all three poliovirus serotypes in both adolescents and adults, with no between-group differences (*p* > 0.05). The GMTs of antibodies after vaccination increased substantially in both groups across all serotypes, reaching 1748.29, 3961.77, and 2089.08 for types 1, 2, and 3 in adolescents, and 1902.58, 4434.39, and 2256.16 in adults, respectively, with no significant between-group differences for any serotype (*p* > 0.05). The GMIs of antibodies after vaccination for poliovirus type 1 were significantly higher in adults than in adolescents (72.12 vs. 28.10, *p* = 0.008); for type 2, the GMIs showed no significant between-group difference (175.85 vs. 147.29, *p* = 0.570); for type 3, adults again showed a significantly higher GMI than adolescents (316.14 vs. 45.56, *p* < 0.001). More details are shown in Table 2.

**Figure 2:**
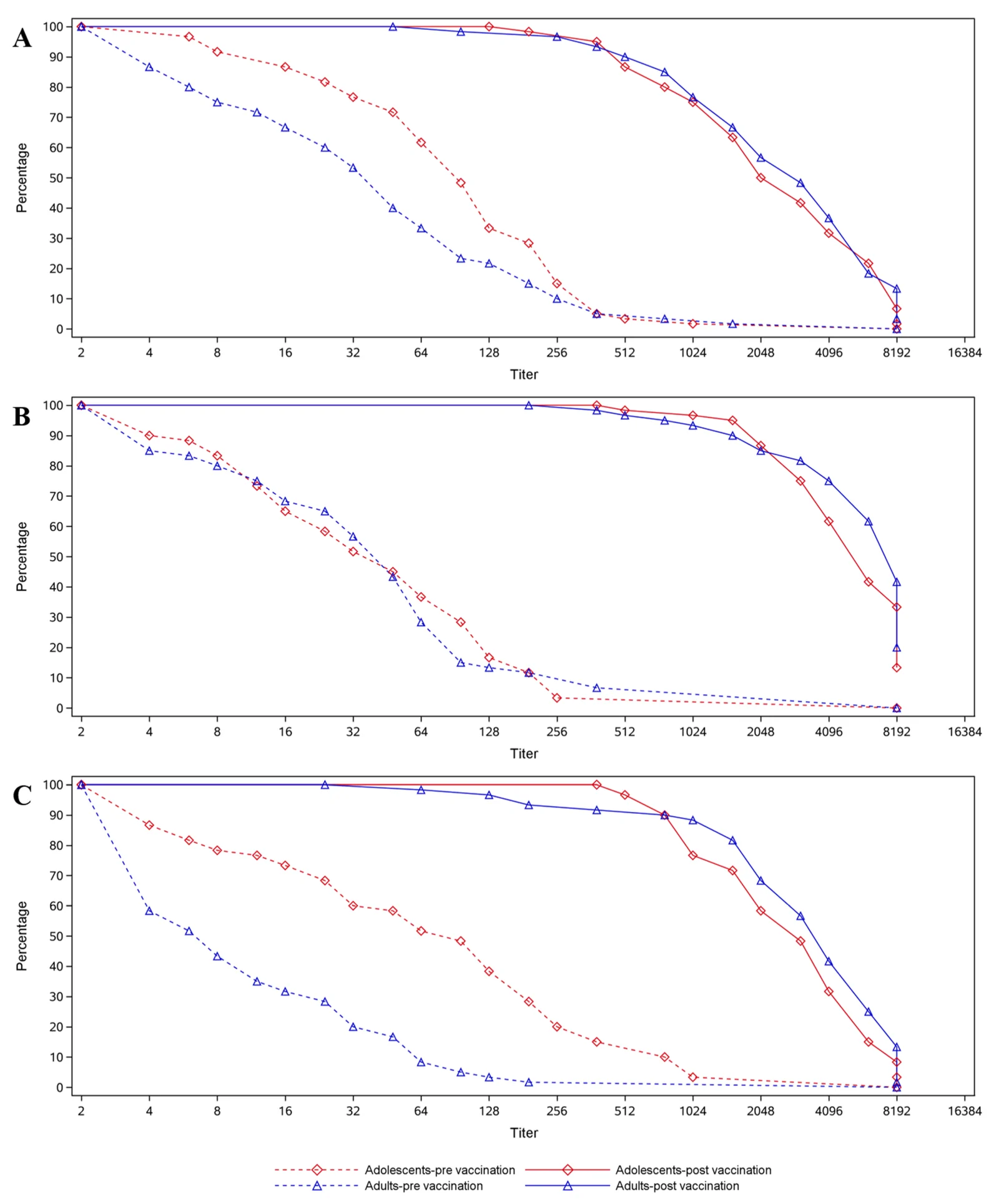
Reverse cumulative distribution curves of neutralising antibody titres against poliovirus types 1, 2, and 3 before and after vaccination in adolescents and adults. (**A**) Poliovirus type 1; (**B**) Poliovirus type 2; (**C**) Poliovirus type 3.

Figure 2 shows the reverse cumulative distribution curves of neutralising antibody titres against poliovirus types 1, 2, and 3 before and 30 days after a single sIPV booster dose, stratified by age group. For all three serotypes, the post-vaccination curves in both age groups shifted substantially rightward relative to their respective pre-vaccination curves, indicating a broad upward redistribution of titres following vaccination. For type 1, the pre-vaccination adolescent curve lay to the right of that of adults, reflecting higher baseline titres in the younger group. After vaccination, both curves converged at high titre levels, with over 95% of participants in each group achieving titres of 1:128 or higher. For type 2, both age groups exhibited comparably high baseline titres, with approximately 80% of participants seropositive before vaccination. Following the booster, the post-vaccination curves for the two age groups were nearly superimposable and positioned at the highest titre ranges among the three serotypes. All participants reached titres of 1:64 or higher, and the vast majority exceeded 1:512, indicating that the type 2 booster response was the strongest and most uniform across age groups. For type 3, the pre-vaccination disparity between age groups was the most pronounced among the three serotypes, with the adolescent curve markedly right-shifted relative to that of adults. After vaccination, however, the two curves converged substantially, with both groups achieving 100% seropositivity and comparable titre distributions. Notably, the adult curve for type 3 underwent the most dramatic rightward shift observed across all serotype and age combinations, reflecting the largest fold increase in titres following booster administration.

### 3.3. Safety

Within 30 days post-vaccination, at least one vaccine-related AE was reported in 21 of 120 participants (17.50%), with similar incidences in the adolescent (10/60, 16.67%) and adult (11/60, 18.33%) groups. Vaccination site pain was the most frequently reported event, occurring in 15 participants (12.50%; 7 adolescents and 8 adults). Other local reactions were uncommon, including swelling in two adults (1.67%) and erythema in one adult (0.83%). All AEs were mild to moderate in severity, and no vaccine-related SAEs were reported in either group. Further details of vaccine-related AEs are presented in Table 3.

## 4. Discussion

The findings of this phase IV open-label trial demonstrate that a single sIPV booster elicits robust neutralising antibody responses against all three poliovirus serotypes in adolescents and adults, with seropositivity rates reaching 100% for all serotypes 30 days post-vaccination. The vaccine was well tolerated, with no vaccine-related SAEs reported. These results provide the first systematic evidence from a clinical study supporting the potential extension of sIPV booster vaccination to adolescent and adult populations in China, an age cohort currently without any recommended poliovirus booster in the national immunisation schedule.

The post-vaccination GMTs observed in both age groups, exceeding 1700 for type 1, 3900 for type 2, and 2000 for type 3, were substantially higher than those reported in infant primary series studies [13,14,16], reflecting the anamnestic nature of the response in individuals with prior vaccination-primed immune systems. This robust boosting effect is consistent with the establishment of durable immunological memory following childhood immunisation, which can be rapidly recalled upon antigen re-exposure [17,18]. Moreover, we observed a notable age-related disparity in baseline seropositivity, particularly for type 3 (78.33% in adolescents vs. 43.33% in adults; *p* < 0.001). This finding aligns with previous seroprevalence data from Italy and Japan, where adolescents demonstrated higher neutralising antibody titres than older adults [11,19], and likely reflects waning antibody levels over time in the absence of natural boosting or scheduled revaccination. A 10-year observational study from Japan further confirmed that while antibodies against types 1 and 2 remained at high levels, some adults showed decreased antibody levels against type 3 [19]. Importantly, despite these baseline differences, the sIPV booster effectively equalised post-vaccination responses across age groups, with post-vaccination GMTs and seropositivity rates becoming statistically indistinguishable between adolescents and adults. The substantially higher GMIs observed in adults, particularly for type 3 (316.14 vs. 45.56; *p* < 0.001), indicate that the booster dose exerts a proportionally greater effect in individuals with lower pre-existing immunity, underscoring its potential value in older populations with waning seroprotection.

Notably, baseline seropositivity for poliovirus type 1 in adults was 75% rather than 100%, suggesting incomplete primary vaccination or waned immunity in a subset of adults. A single booster closed this observed immunity gap across all participants. Thus, despite substantial baseline seropositivity, a booster dose may remain relevant for some adults. The low baseline titres against poliovirus type 3 in adults (GMT, 7.14, compared with 45.85 in adolescents) may reflect historically weaker type 3 priming after trivalent OPV and more pronounced antibody waning over time [19,20]. A supplemental-dose study likewise reported a comparatively weaker type 3 response after trivalent OPV-based priming [20]. However, our data cannot distinguish these explanations from age-related waning, differences in individual vaccination history, or prior poliovirus exposure.

From a mechanistic perspective, the brisk anamnestic response observed in our study, with substantial antibody rises within 30 days, is consistent with recall of poliovirus-specific immune memory established during childhood immunisation [21]. Upon antigen re-exposure, memory B cells can proliferate and differentiate into antibody-secreting plasma cells [22], and long-lived plasma cells may contribute to persistent antibody production [23]. Nevertheless, serological waning should not be interpreted as proof of absent immune memory or protection, and the relationship between circulating antibody titres and protection requires cautious interpretation [24]. Comparisons between Sabin- and Salk-strain IPV also require caution because the vaccines differ in seed strains and antigenic structure, and measured neutralising titres may depend on the virus strain used in the assay [25]. Our study included no Salk-IPV comparator and used Sabin strains in the neutralisation assay; therefore, the results demonstrate a strong homologous response to the sIPV used here but do not establish equivalence or superiority relative to Salk-IPV. Studies from Cuba and Japan have similarly shown that an IPV booster can increase poliovirus neutralising antibody levels in adults [7,8].

Whether sIPV-elicited antibodies neutralise wild-type polioviruses is clinically relevant because wild-type poliovirus type 1 (WPV1) remains endemic in countries neighbouring China. A cross-neutralisation analysis of sera from 250 infants who had received the same Sinovac sIPV found neutralising activity against Sabin, Salk, and recently circulating poliovirus strains, including a Xinjiang WPV1 isolate [15]. Because these data were generated in infants and were not obtained from a randomised adult sIPV-versus-Salk-IPV booster comparison, they provide supportive but indirect evidence regarding the breadth of the antibody response and do not establish equivalence or superiority between sIPV and Salk-IPV in the adult booster setting.

The present trial assessed immunogenicity only through 30 days and therefore does not establish the durability of the booster response in adolescents or adults. As supportive context, a phase IV follow-up of children who completed a four-dose sIPV series at 2, 3, 4, and 18 months reported seropositivity above 99% for all three serotypes five years after completion of vaccination [26]. This finding supports substantial antibody persistence after a paediatric four-dose regimen; however, it cannot be extrapolated directly to the durability of a single booster in previously vaccinated adolescents or adults. Longer follow-up studies in older age groups are needed.

Our safety findings align with the established tolerability profile of sIPV. The overall vaccine-related AE rate of 17.50% and the predominance of mild-to-moderate injection-site pain (12.50%) are consistent with previous phase II and III trials of sIPV in infants and children [1,13,14,15], as well as adult IPV booster studies from Japan [8]. The absence of vaccine-related SAEs and the low frequency of systemic reactions support the favourable safety profile of a single sIPV booster in these age groups. From a manufacturing perspective, sIPV uses attenuated Sabin strains and is associated with lower biosafety risks than conventional IPV production based on virulent wild-type strains, which requires higher-containment facilities [27,28,29]. This feature may facilitate broader and more secure IPV production in the polio endgame.

Although adolescents and adults are not routine target populations for poliovirus vaccination, targeted IPV use remains programmatically important in these age groups. WHO’s Global Action Plan for Poliovirus Containment (GAPIV) mandates that all personnel working in poliovirus-essential facilities must be appropriately immunised and screened as a core biosafety requirement [30], and national catch-up vaccination guidance identifies continuous strategies as essential for maintaining age-appropriate immunity [31]. As the GPEI progresses toward the post-eradication era and countries transition from OPV-containing to IPV-only schedules, the need for booster vaccination in older age groups becomes increasingly relevant. Although low antibody titres in adults may not alone justify universal boosters if memory immunity remains adequate, mathematical modelling suggests that, in the worst-case scenario, approximately 10% of individuals receiving five IPV doses could be seronegative to each serotype by adulthood [32]. The detection of circulating vaccine-derived poliovirus type 2 (cVDPV2) in multiple high-income countries between 2022 and 2024 further underscores the persistent importation risk [33]. By demonstrating robust neutralising antibody responses and acceptable tolerability of a single sIPV booster in both adolescents and adults, our study provides clinically relevant evidence supporting occupational-risk vaccination, catch-up immunisation, and potential broader booster strategies to sustain population immunity in the post-eradication era.

## 5. Conclusions and Limitations

This study has several limitations. First, the sample size of 60 participants per group, while statistically justified and sufficient for the primary immunogenicity endpoints, may limit the detection of rare safety events, the precision of subgroup estimates, and the generalisability of our findings to the broader adolescent and adult populations, particularly given the heterogeneity in baseline immunity arising from unverified individual vaccination histories and potential prior poliovirus exposure. Second, the open-label, single-arm design precludes direct comparison with a control group receiving placebo or alternative vaccine, and the lack of long-term follow-up beyond 30 days restricts conclusions about the durability of the booster-induced antibody response. Third, individual-level poliovirus vaccination histories were not systematically collected or verified. Given enrolment in 2025 and participant ages of 7 to 50 years, the study population comprised birth cohorts from approximately 1975 to 2018. Most adults and older adolescents would generally have been eligible for earlier OPV-based programmes, whereas some of the youngest adolescents may have received the sequential schedule introduced in May 2016, consisting of one IPV dose followed by three bOPV doses. None of the participants would have been routinely primed as infants under the two-IPV plus two-bOPV schedule introduced nationally in late 2019, although subsequent catch-up vaccination or privately obtained IPV cannot be excluded [6,34]. Participants may therefore have differed in vaccine formulation, number and timing of doses, and opportunities for vaccine-virus or natural boosting. This unmeasured heterogeneity could have contributed to the observed baseline titre differences, particularly the lower type 3 seropositivity in adults, and prevents us from separating age-related waning from effects related to the primary schedule or number of prior doses. It also limits schedule-specific estimates of booster benefit and the identification of subgroups most likely to require catch-up vaccination. Nevertheless, the uniformly high post-vaccination seropositivity across age groups supports the overall conclusion of strong short-term immunogenicity, while decisions on targeted boosting or catch-up should be informed by future studies with verified vaccination records and analyses stratified by prior regimen. Fourth, the absence of a comparator group receiving Salk-strain IPV limits our ability to comment on potential differences between Sabin- and Salk-strain vaccines in the adult booster setting.

In conclusion, a single booster dose of sIPV induced robust neutralising antibody responses against all three poliovirus serotypes in both adolescents (7 to 17 years) and adults (18 to 50 years), with post-vaccination seropositivity rates reaching 100% across all serotypes and age groups. The vaccine demonstrated a favourable safety profile, with no vaccine-related serious adverse events and a predominance of mild-to-moderate injection-site pain. Notably, adults with lower baseline immunity derived proportionally greater fold-increases in antibody titres, highlighting the potential benefit of booster vaccination in older populations with waning seroprotection. These findings provide the first clinical evidence supporting the potential extension of sIPV booster use to adolescent and adult populations in China, where no such recommendation currently exists, and align with international efforts to maintain population immunity in the polio post-eradication era. Future studies with extended follow-up and larger sample sizes are warranted to assess the long-term persistence of booster-induced immunity and to evaluate the optimal timing and frequency of adult revaccination strategies, as well as the potential impact of such booster programmes on population-level herd immunity.

## Figures and Tables

**Table 1:** Baseline demographic and clinical characteristics of study participants.

Variables	Total (*N* = 120)	Adolescents (*N* = 60)	Adults (*N* = 60)
Gender, *n* (%)			
Male	49 (40.83)	30 (50.00)	19 (31.67)
Female	71 (59.17)	30 (50.00)	41 (68.33)
Age, Mean (SD)	23.5 (14.6)	10.0 (2.5)	37.0 (7.4)
Ethnicity, *n* (%)			
Han	118 (98.33)	59 (98.33)	59 (98.33)
Others	2 (1.67)	1 (1.67)	1 (1.67)
Height (cm), Mean (SD)	154.95 (15.71)	144.94 (15.42)	164.96 (7.48)
Weight (kg), Mean (SD)	55.39 (21.11)	41.12 (15.64)	69.65 (15.49)
Armpit temperature (°C), Mean (SD)	36.41 (0.28)	36.41 (0.31)	36.41 (0.25)

**Table 2:** Immunogenicity outcomes for poliovirus types 1, 2, and 3 in adolescents and adults.

Immunogenicity	Total (*N* = 120)	Adolescents (*N* = 60)	Adults (*N* = 60)	*p* Value
Poliovirus type 1				
Seroconversion rate				0.543 ^a^
*n* (%)	108 (90%)	53 (88.33)	55(91.67)	
95%CI	83.18, 94.73	77.43, 95.18	81.61, 97.24	
Seropositivity rate				
Pre–immunization				0.014 ^a^
*n* (%)	100 (83.33)	55 (91.67)	45 (75.00)	
95%CI	75.44, 89.51	81.61, 97.24	62.14, 85.28	
Post–immunization				0.999 ^b^
*n* (%)	120 (100)	60 (100)	60 (100)	
95%CI	96.97, 100	94.04, 100	94.04, 100	
GMT (95%CI)				
Pre–immunization	40.51 (30.61, 53.61)	62.21 (44.17, 87.62)	26.38 (17.23, 40.38)	0.002 ^c^
Post–immunization	1823.81 (1493.50, 2227.17)	1748.29 (1323.73, 2309.03)	1902.58 (1417.00, 2554.55)	0.677 ^c^
GMI (95%CI)	45.02 (31.61, 64.11)	28.10 (17.01, 46.41)	72.12 (44.51, 116.86)	0.008 ^c^
Poliovirus type 2				
Seroconversion rate (95%CI)				0.999 ^a^
*n* (%)	118 (98.33)	59 (98.33)	59 (98.33)	
95%CI	94.11, 99.80	91.06, 99.96	91.06, 99.96	
Seropositivity rate (95%CI)				
Pre–immunization				0.637 ^a^
*n* (%)	98 (81.67)	50 (83.33)	48 (80.00)	
95%CI	73.57, 88.14	71.48, 91.71	67.67, 89.22	
Post–immunization				0.999 ^b^
*n* (%)	120 (100)	60 (100)	60 (100)	
95%CI	96.97, 100	94.04, 100	94.04, 100	
GMT (95%CI)				
Pre–immunization	26.04 (20.07, 33.80)	26.90 (18.67, 38.75)	25.22 (17.20, 36.97)	0.808 ^c^
Post–immunization	4191.42 (3630.57, 4838.91)	3961.77 (3284.74, 4778.34)	4434.39 (3550.20, 5538.80)	0.440 ^c^
GMI (95%CI)	160.94 (118.43, 218.71)	147.29 (93.40, 232.27)	175.85 (115.18, 268.48)	0.570 ^c^
Poliovirus type 3				
Seroconversion rate (95%CI)				0.032 ^a^
*n* (%)	111 (92.50)	52 (86.67)	59 (98.33)	
95%CI	86.24, 96.51	75.41, 94.06	91.06, 99.96	
Seropositivity rate (95%CI)				
Pre–immunization				<0.001 ^a^
*n* (%)	73 (60.83)	47 (78.33)	26 (43.33)	
95%CI	51.50, 69.61	65.80, 87.93	30.59, 56.76	
Post–immunization				0.999 ^b^
*n* (%)	120 (100)	60 (100)	60 (100)	
95%CI	96.97, 100	94.04, 100	94.04, 100	
GMT (95%CI)				
Pre–immunization	18.09 (12.86, 25.44)	45.85 (28.15, 74.68)	7.14 (5.02, 10.15)	<0.001 ^c^
Post–immunization	2171.01 (1793.65, 2627.77)	2089.08 (1671.56, 2610.89)	2256.16 (1643.79, 3096.66)	0.692 ^c^
GMI (95%CI)	120.02 (80.54, 178.85)	45.56 (25.13, 82.60)	316.14 (208.27, 479.88)	<0.001 ^c^

^a^ Chi-square test. ^b^ Fisher’s exact test. ^c^ Independent-samples t-test.

**Table 3:** Adverse events reported within 30 days post–vaccination.

Adverse Events	Total (*N* = 120)	Adolescents (*N* = 60)	Adults (*N* = 60)
Vaccine-related AEs, *n* (%)	21 (17.50)	10 (16.67)	11 (18.33)
General disorders and administration site conditions	16 (13.33)	8 (13.33)	8 (13.33)
Vaccination site swelling	2 (1.67)	0	2 (3.33)
Pyrexia	1 (0.83)	1 (1.67)	0
Vaccination site pain	15 (12.50)	7 (11.67)	8 (13.33)
Vaccination site erythema	1 (0.83)	0	1 (1.67)
Gastrointestinal disorders	1 (0.83)	0	1 (1.67)
Nausea	1 (0.83)	0	1 (1.67)
Metabolism and nutrition disorders	1 (0.83)	0	1 (1.67)
Decreased appetite	1 (0.83)	0	1 (1.67)
Respiratory, thoracic and mediastinal disorders	5 (4.17)	4 (6.67)	1 (1.67)
Cough	5 (4.17)	4 (6.67)	1 (1.67)
Rhinorrhoea	2 (1.67)	2 (3.33)	0
Oropharyngeal pain	2 (1.67)	1 (1.67)	1 (1.67)
Nervous system disorders	3 (2.50)	2 (3.33)	1 (1.67)
Headache	3 (2.50)	2 (3.33)	1 (1.67)
Skin and subcutaneous tissue disorders	1 (0.83)	0	1 (1.67)
Rash	1 (0.83)	0	1 (1.67)

## Data Availability

Restrictions apply to the datasets. The datasets presented in this article are not readily available because the data are proprietary to the study sponsor, Sinovac Biotech Ltd., and restrictions apply to their sharing. Requests to access the datasets should be directed to the corresponding authors and will be evaluated in accordance with the sponsor’s data sharing policy.
